# The Gray Area of Sarcoidosis and Tuberculosis: A Diagnostic Enigma

**DOI:** 10.7759/cureus.71763

**Published:** 2024-10-18

**Authors:** Shailesh B Meshram, Rhea P Gandhi, Geedhara Harsha Vardhan Reddy

**Affiliations:** 1 Respiratory Medicine, Dr. D. Y. Patil Medical College, Hospital & Research Centre, Dr. D. Y. Patil Vidyapeeth (Deemed to be University), Pune, IND

**Keywords:** granulomas, histopathology, inflammation, lung, lymphadenopathy, mycobacterium, radiology, sarcoidosis, steroids, tuberculosis

## Abstract

This case report presents a challenging diagnostic enigma, where a 29-year-old male patient presented with symptoms and signs favoring both tuberculosis (TB) and sarcoidosis. The patient’s plain chest radiograph showed bilateral hilar opacities, while a contrast-enhanced computed tomography (CECT) scan revealed multiple mediastinal and hilar lymph nodes. Histopathology of a mediastinal lymph node biopsy showed necrotizing granulomatous inflammation, favoring TB. However, the patient’s symptoms did not resolve with antitubercular treatment, and further investigations revealed a working diagnosis of pulmonary sarcoidosis. The patient’s symptoms improved with corticosteroid therapy, and subsequent plain chest radiographs and high-resolution computed tomography (HRCT) scans showed near complete resolution of lung infiltrates. This case highlights the importance of careful evaluation and interpretation of biopsy results and the use of advanced diagnostic techniques to diagnose and differentiate between TB and sarcoidosis accurately.

## Introduction

The differential diagnosis between tuberculosis (TB) and sarcoidosis presents a significant challenge in clinical practice due to overlapping clinical and radiographic features. Both conditions can manifest with similar symptoms, such as cough, fever, and weight loss, and can affect multiple organ systems, including the lungs. Sarcoidosis is a systemic granulomatous disease characterized by non-caseating granulomas, while TB is a bacterial infection caused by *Mycobacterium tuberculosis* that leads to caseating granulomas.

The diagnostic complexity arises from the similarities in radiographic findings and the potential for both conditions to present with pulmonary infiltrates. For instance, both can show bilateral hilar lymphadenopathy and pulmonary infiltrates on plain chest radiographs, complicating the initial assessment [1]. Additionally, both diseases can present with elevated serum angiotensin-converting enzyme (ACE) levels, a marker often associated with sarcoidosis but not exclusively so [2]. Furthermore, the immunological and histopathological overlap requires careful interpretation of biopsy results and advanced diagnostic techniques to reach an accurate diagnosis. Recent advances in diagnostic modalities, including molecular techniques like polymerase chain reaction (PCR) for detecting *M. tuberculosis* deoxyribonucleic acid (DNA) and newer imaging techniques, have improved diagnostic accuracy [3,4]. Understanding the nuances of these diagnostic challenges is essential as the treatments for TB and sarcoidosis differ significantly. We present a case of a young male presenting with symptoms and signs favoring TB as well as sarcoidosis.

## Case presentation

A 29-year-old male chef and smoker presented with chief complaints of cough for a year, which was dry in nature without any specific triggers and no hemoptysis, and breathlessness for a year of modified Medical Research Council (mMRC) grade one with occasional episodes of wheezing. He also gave a history of intermittent episodes of low-grade fever for a year with loss of weight and appetite. The patient had been admitted to a private hospital a year back with these complaints of 2 weeks duration and was empirically started on intravenous antibiotics (Piperacillin-Tazobactam) for suspected bacterial infection. However, his sputum studies for Gram stain, Ziehl-Neelsen (ZN) stain, aerobic culture, and cartridge-based nucleic acid amplification test (CBNAAT) were inconclusive.

**Figure 1:**
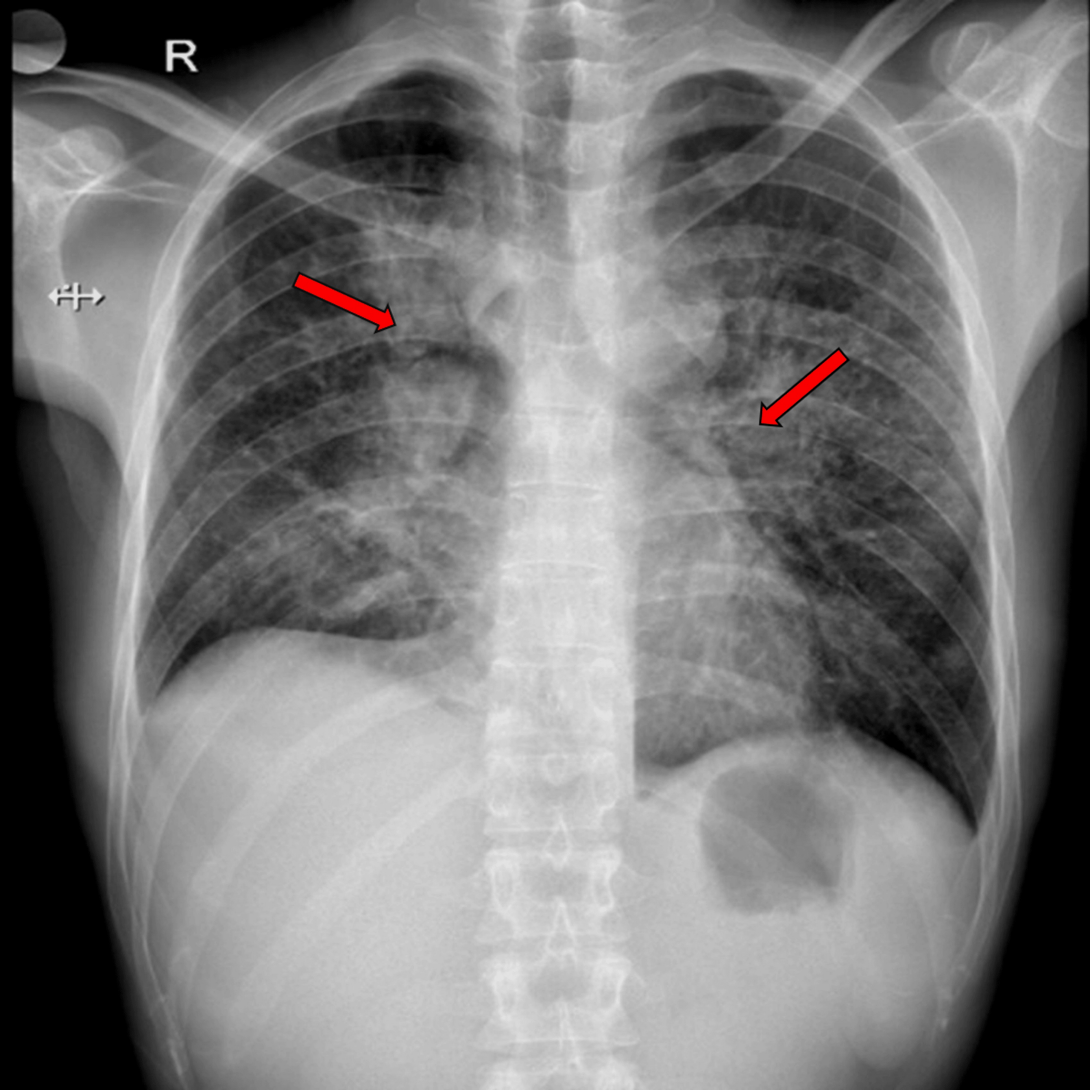
Plain chest radiograph on presentation showing bilateral hilar opacities (red arrows).

His plain chest radiograph revealed bilateral hilar and parenchymal opacities (Figure 1), which was followed by a contrast-enhanced computed tomography (CECT) of the thorax that showed multiple, peripherally enhancing, conglomerate, necrotic, mediastinal, and hilar lymph nodes, the largest measuring 3.9 by 2.9 cm. There were also focal areas of ground-glass opacities and interlobular septal thickening involving lung parenchyma in the peri-hilar region.

**Figure 2:**
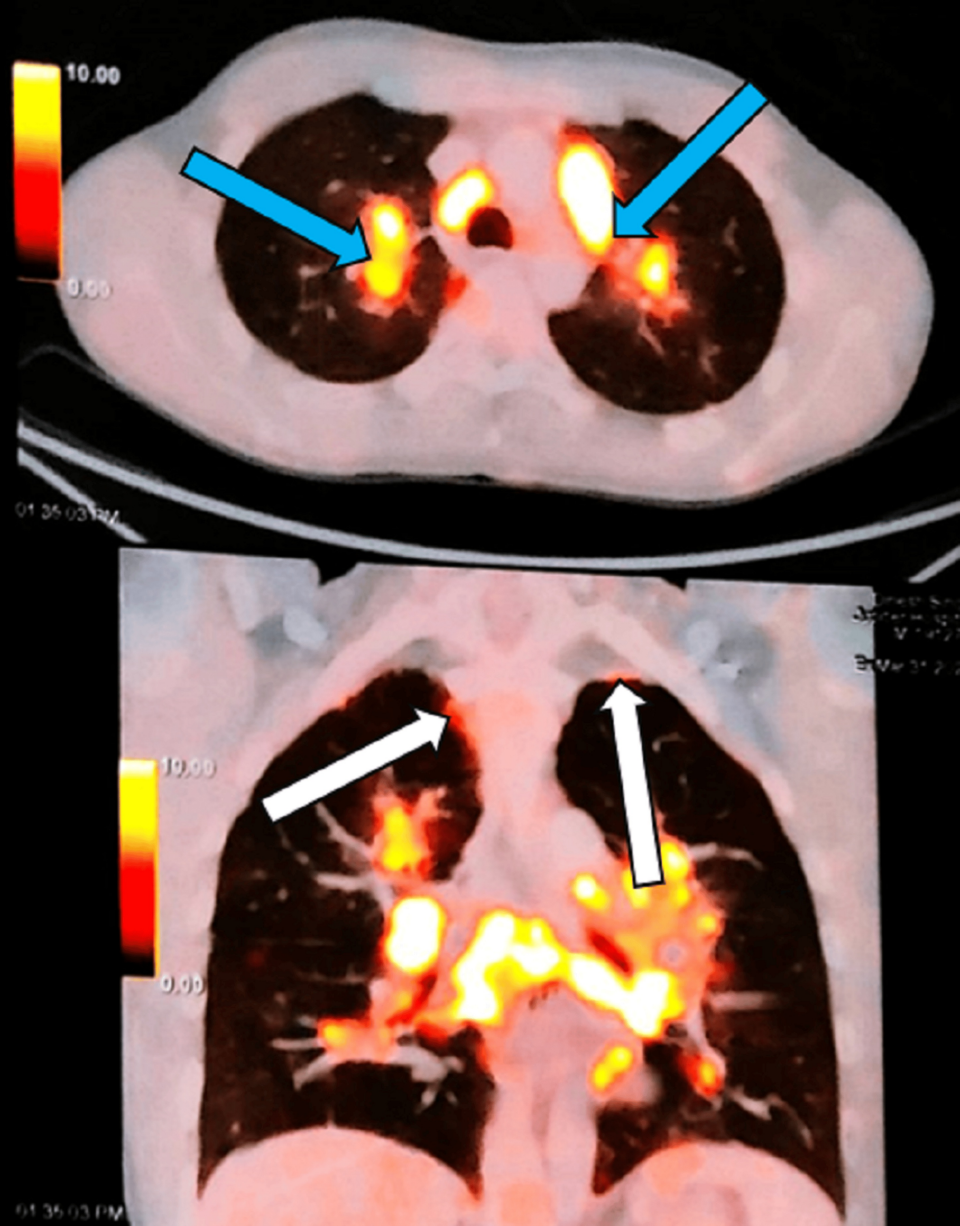
PET-CT scan showing metabolically active, multiple mediastinal, and bilateral hilar lymphadenopathy (blue arrows) and metabolically active bilateral pleural nodules (white arrows). PET-CT: positron emission tomography-computed tomography

A positron emission tomography-computed tomography (PET-CT) scan was done, which showed metabolically active, multiple mediastinal, and bilateral hilar lymphadenopathy with contiguous extension in the upper lobe of both lungs. Additionally, there were metabolically active bilateral upper pleural nodules (Figure 2).

A CT-guided biopsy of the mediastinal lymph node was performed, and the histopathology report showed necrotizing granulomatous inflammation, with epithelioid cells and lymphocytes, without caseous necrosis, which favored TB. The patient was advised to take tablet rifampicin (R) 600 mg once daily (OD), isoniazid (H) 300 mg OD, pyrazinamide (Z) 1500 mg OD, ethambutol (E) 800 mg OD for 3 months, followed by HRE for 6 months, and finally HR for one more month. The total duration of treatment was 10 months. However, the patient’s symptoms did not resolve, and another sputum CBNAAT was repeated, which was negative.

**Figure 3:**
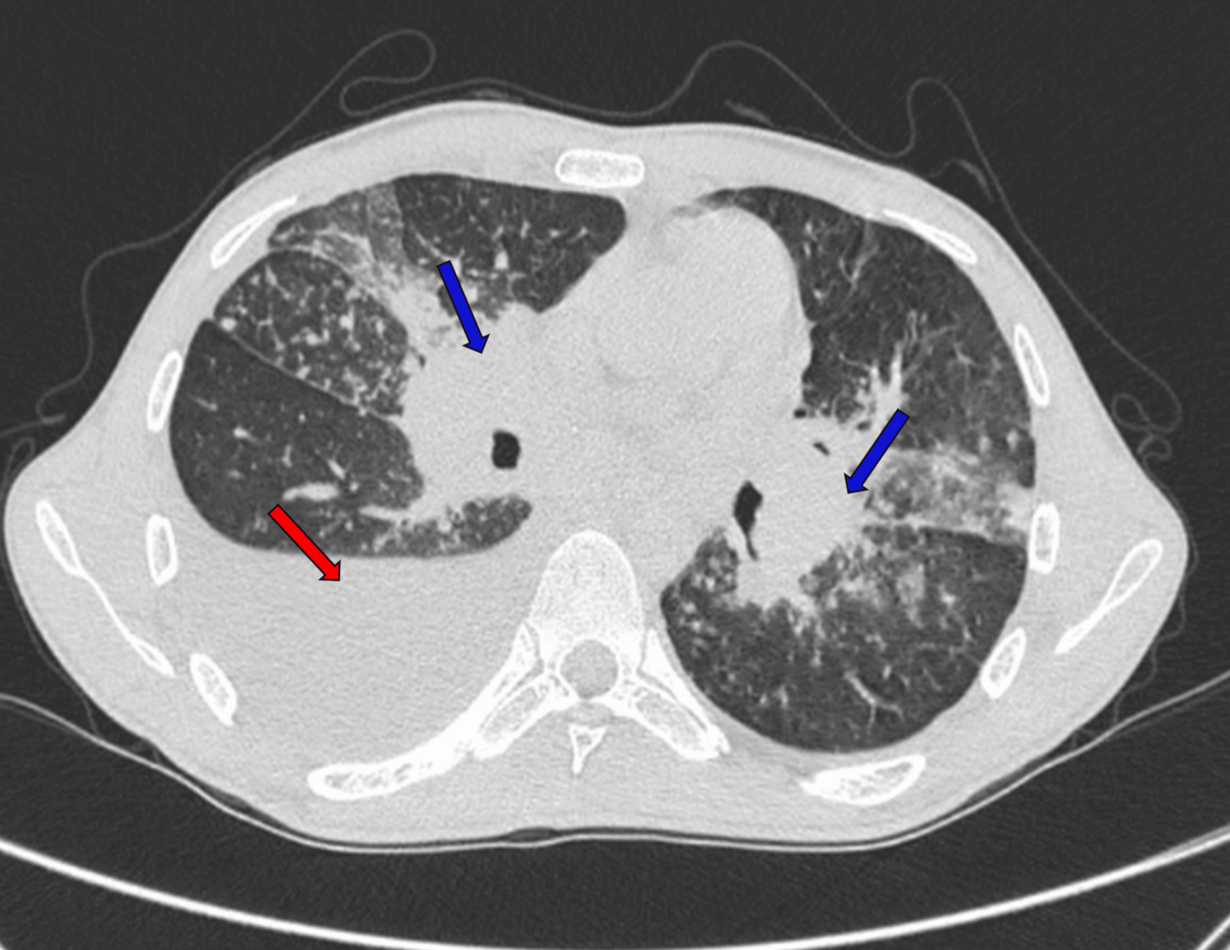
CECT thorax showing right moderate pleural effusion (red arrow) with mediastinal shift to the left side, along with dense consolidations (blue arrows). CECT: contrast-enhanced computed tomography

At this time, the patient presented to our hospital, and a CECT thorax was done, which showed right moderate pleural effusion with mediastinal shift to the left side, along with dense consolidation and fibro-atelectatic bands, centrilobular nodules and diffuse tree-in-bud appearance bilaterally (Figure 3).

**Figure 4:**
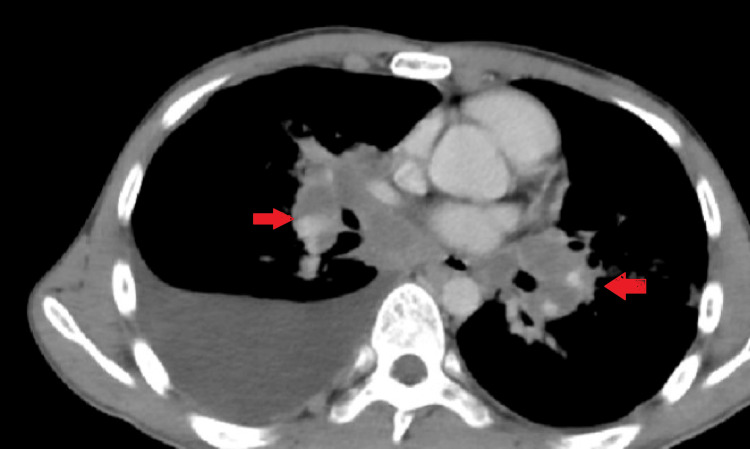
Mediastinal window of CECT thorax showing bilateral hilar lymphadenopathy (red arrows). CECT: contrast-enhanced computed tomography

There were multiple peripherally enhancing, conglomerated lymph nodes with central necrosis in the bilateral hilar regions, the largest measuring 35 x 37 mm on the right side with mediastinal lymphadenopathy (Figure 4). Thick fibrotic infiltrates with volume loss were noted in the lateral segment of the right middle lobe. Ground-glass opacities were noted in bilateral perihilar regions and lingula.

**Figure 5:**
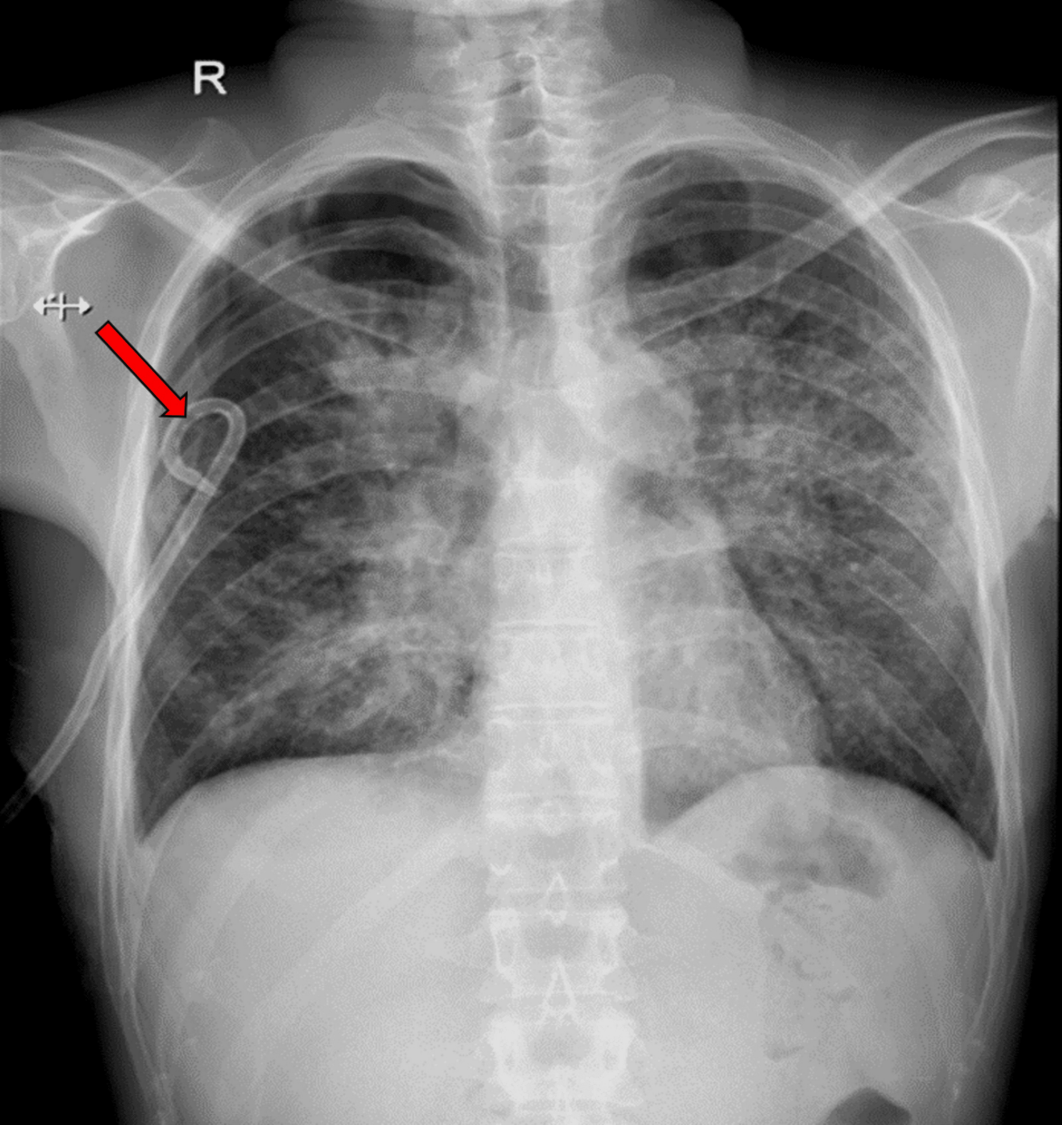
Plain chest radiograph showing right-sided pigtail in situ (red arrow) for draining pleural effusion.

A right-sided tube thoracostomy was performed (Figure 5) to drain the effusion which was exudative and lymphocytic-predominant with an adenosine deaminase (ADA) level of 39.7 units/L.

**Figure 6:**
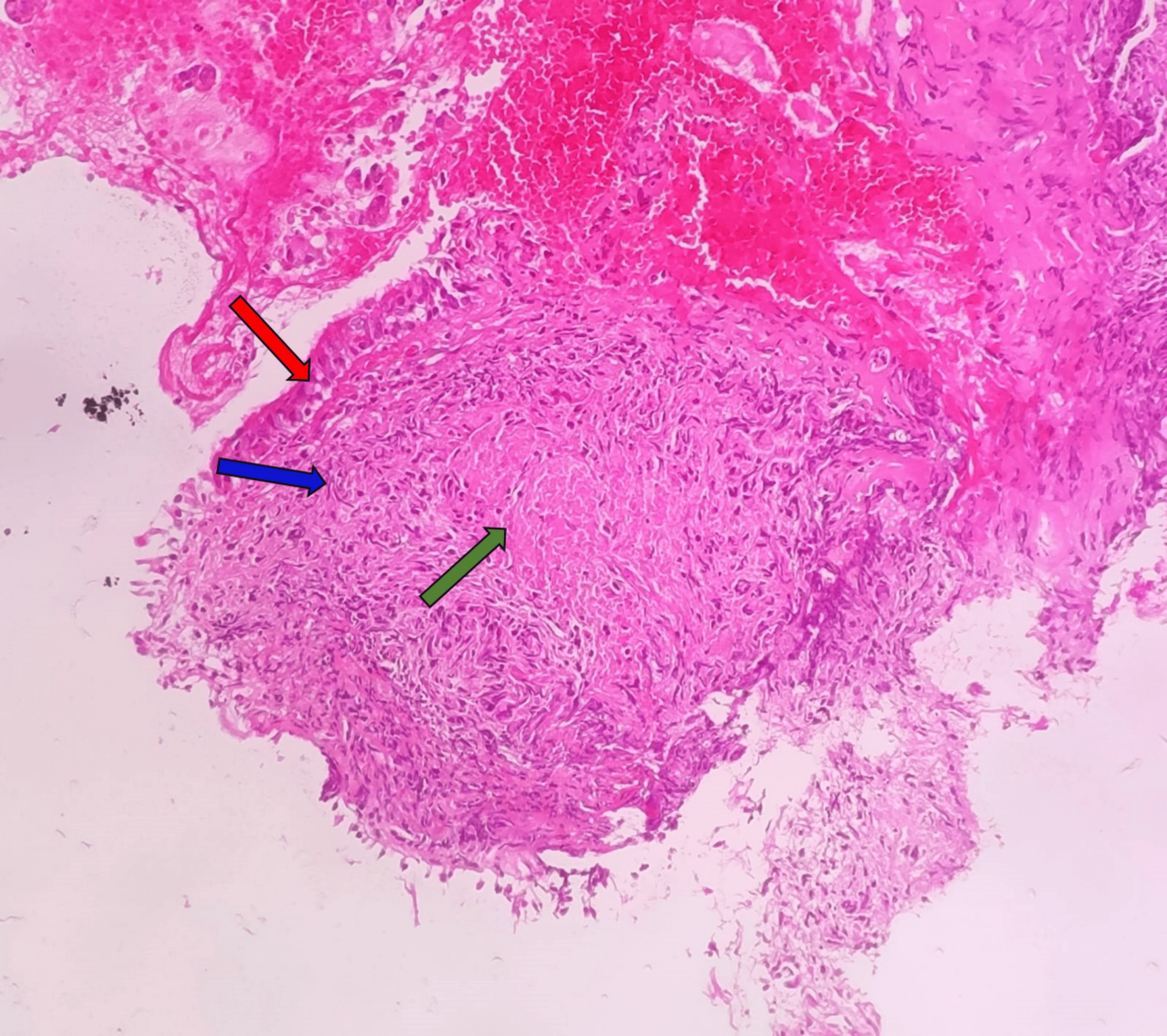
HPE of EBB showing bronchial lining (red arrow) and granuloma (blue arrow) with central necrosis (green arrow). HPE: histopathological examination; EBB: endobronchial biopsy

CBNAAT and malignant cytology of pleural fluid and bronchoalveolar lavage (BAL) were negative. No acid-fast bacilli (AFB) were seen on ZN staining, and the culture showed no growth. No secretions or endobronchial masses were seen during the bronchoscopy. The endobronchial biopsy (EBB) was taken from the medial basal, lateral basal, and posterior basal segmental bronchi of the right lower lobe. EBB showed large, confluent granulomas epithelioid cells and Langhans’ giant cells. An occasional granuloma showed focal necrosis (Figure 6).

There was no evidence of fungal elements or neoplastic cells. ZN stain was negative for AFB. Transbronchial lung biopsy (TBLB) showed a single fragment of alveolated lung parenchyma with focal fibrosis without granulomas or neoplastic cells. Transbronchial needle aspiration (TBNA) of subcarinal lymph nodes predominantly consisted of short bronchial epithelial cells without granulomas. Since there were necrotizing granulomas seen on histopathological examination, the findings favored TB over sarcoidosis. The histopathological findings were consistent with TB, but a working diagnosis of pulmonary sarcoidosis was made clinico-radiologically. Ultrasonography (USG) of the abdomen and pelvis showed mesenteric and retro-peritoneal lymphadenopathy. Examination of the eyes by an ophthalmologist showed no signs of sarcoidosis. Serum ACE and urinary calcium levels were normal. Interferon-gamma release assay (IGRA) and Mantoux tests were negative. Spirometry showed restriction and severe obstruction without post-bronchodilator reversibility (Table 1).

**Table 1:** Baseline spirometry result. mcg: microgram; FVC: forced vital capacity; L: liters; FEV1: forced expiratory volume in the first second; FEF 25-75%: forced expiratory flow at 25-75%; L/s: liters per second

Values	Pre	% predicted (pre)	Post Salbutamol (400 mcg)	% predicted (post)
FVC (L)	2.37	55	2.58	60
FEV1 (L)	1.72	47	1.85	50
FEV1/FVC (%)	72.3	87	71.9	87
FEF 25-75% (L/s)	1.18	33	1.44	40

The patient was started on tablet methylprednisolone 32 mg OD and inhalational bronchodilators for a month, after which he showed clinical improvement. Further, the steroid dosage was tapered, and a repeat plain chest radiograph showed the resolution of the bilateral infiltrates. Serial spirometry tests showed improvement in lung function values (Table 2).

**Table 2:** Serial spirometry reports after starting treatment with steroids. Post-bronchodilator test was not done as there was no obstruction. FVC: forced vital capacity; L: liters; FEV1: forced expiratory volume in the first second; FEF 25-75%: forced expiratory flow at 25-75%; L/s: liters per second.

After Treatment	2 Months		3 Months		4 months	
Values	Pre	% predicted (pre)	Pre	% predicted (pre)	Pre	% predicted (pre)
FVC (L)	2.51	59	2.76	64	2.78	67
FEV1 (L)	2.37	65	2.5	68	2.46	69
FEV1/FVC	94.5	114	90.5	109	88.2	107
FEF 25-75% (L/s)	2.9	81	2.66	74	2.5	71

**Figure 7:**
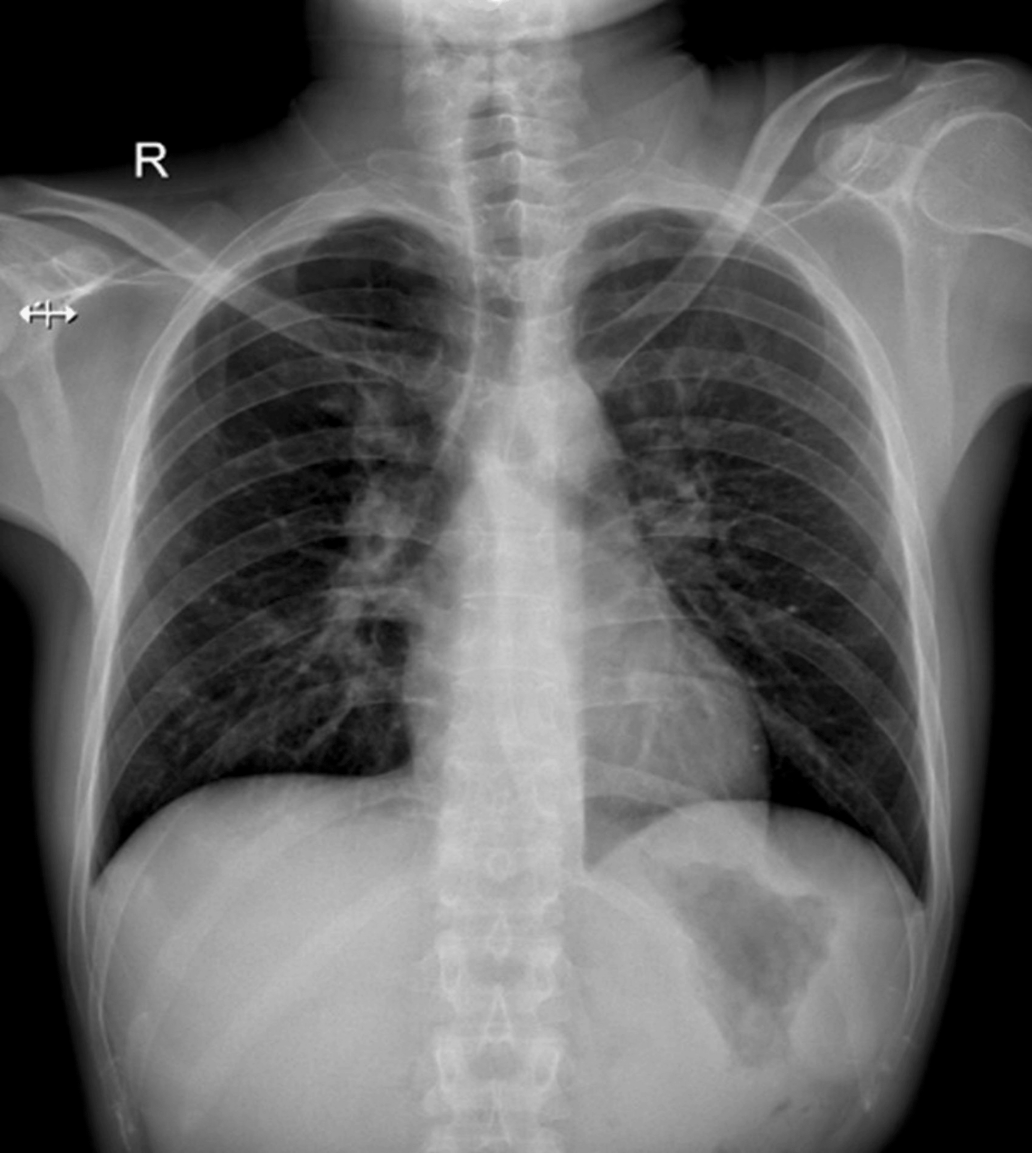
Plain chest radiograph showing near complete resolution of bilateral hilar opacities, post 4 months of steroid treatment.

**Figure 8:**
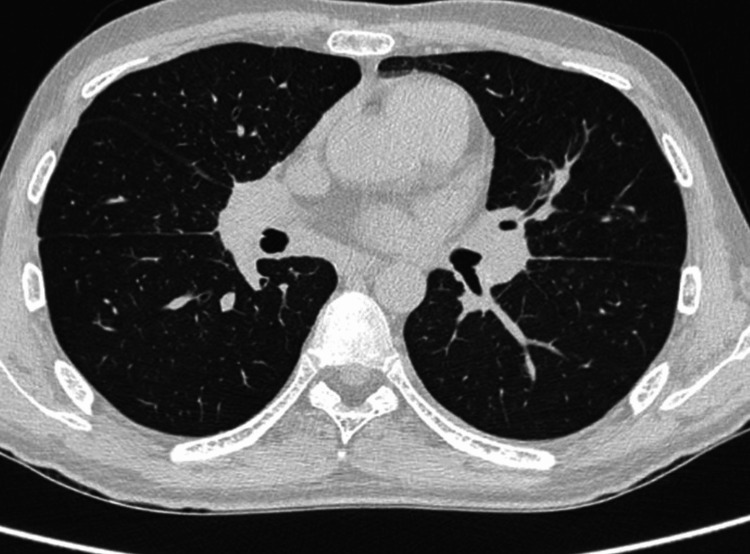
HRCT thorax post 4 months of steroid treatment. HRCT: high-resolution computed tomography

After 4 months of steroid treatment, a radiograph (Figure 7) and high-resolution computed tomography (HRCT) scan of the chest (Figure 8) showed near complete resolution of the consolidation, fibro-atelectatic bands, and centrilobular nodules. This improvement, hence, confirmed our diagnosis of sarcoidosis. The patient has been continued on tablet methylprednisolone 4 mg twice a day (BD) with regular follow-up.

## Discussion

Pulmonary sarcoidosis is a systemic disease marked by non-caseating granulomas primarily affecting the lungs, though it can also involve other organs. Clinically, sarcoidosis often presents without symptoms and is discovered incidentally through chest radiographs. When symptoms do occur, they can include cough, difficulty breathing, and chest pain. However, TB often includes systemic symptoms like fever, night sweats, and weight loss, which are less commonly associated with sarcoidosis [5]. The pathophysiology of sarcoidosis involves a complex interaction between genetic predisposition and environmental factors. Genetic links have been identified, particularly related to certain human leukocyte antigen (HLA) types [6]. Environmental exposures, including dust, mold, or infections, are suspected triggers, although the precise antigens involved are still unknown. The condition is thought to result from an exaggerated type-1 T-helper (Th1-type) immune response, leading to the formation of granulomas due to the accumulation of macrophages and other immune cells [7].

Diagnosis usually involves imaging techniques like plain chest radiographs or HRCT, which reveal typical findings such as bilateral hilar lymphadenopathy and reticular opacities in sarcoidosis. Pulmonary nodules, mainly with perilymphatic distribution and along interlobular septations are seen. Cystic lung changes, which are typically seen in conditions like Langerhans cell histiocytosis (LCH), can also appear in sarcoidosis, particularly in advanced stages. Pulmonary vasculature involvement can present as pulmonary artery aneurysms or stenosis, detectable through imaging techniques such as pulmonary angiography or magnetic resonance imaging (MRI). Vascular changes may contribute to symptoms like hemoptysis or pulmonary hypertension. Bronchiectasis can develop, particularly in patients with long-standing disease. While not a common feature, pleural effusion in sarcoidosis can complicate the clinical picture, as seen in our patient, and contribute to symptoms such as dyspnea and pleuritic chest pain. Sarcoid granulomas can occasionally be found in atypical locations within the lungs, such as within the airways or pleura [8-13]. These unusual sites can make diagnosis challenging and may require specialized biopsy techniques for accurate identification. TB often shows upper lobe infiltrates, cavitary lesions, and bronchogenic spread [14].

Confirmatory diagnosis is made via biopsy, where non-caseating granulomas are observed in sarcoidosis in affected tissues like lymph nodes or lung parenchyma, whereas TB features caseating granulomas. This distinction is essential but can sometimes be difficult if the granulomas are poorly formed or if there is an atypical presentation [15]. Microbiological testing is essential for diagnosing TB. Tests include sputum smear for AFB and cultures for *M. tuberculosis*. Ensuring that TB is ruled out through these tests is vital to avoid misdiagnosis [16]. Recent advancements in serological and molecular diagnostics, such as IGRAs, have improved the ability to distinguish between sarcoidosis and TB. IGRAs can help identify latent TB infection, aiding in the exclusion of TB in patients with suspected sarcoidosis [17]. However, these tests might not differentiate between active TB and sarcoidosis. The location of granulomas can provide diagnostic clues. TB granulomas are often confined to the lung parenchyma, whereas sarcoid granulomas can be found in various organs beyond the lungs. Extrapulmonary involvement in sarcoidosis may offer additional diagnostic information [18].

Treatment for pulmonary sarcoidosis depends on symptom severity and disease progression. Asymptomatic patients may not need immediate treatment but should be monitored regularly. For those with significant symptoms or progressive disease, corticosteroids like prednisone are commonly used to reduce inflammation and alleviate respiratory symptoms [19]. In cases where corticosteroids are ineffective or unsuitable, other immunosuppressive drugs, such as methotrexate or azathioprine, may be considered [20].

The prognosis of sarcoidosis can vary. Many patients experience resolution of symptoms without long-term issues, while others may develop chronic disease with potential complications such as pulmonary fibrosis and reduced lung function. Prognosis is influenced by factors like disease stage at diagnosis and response to treatment. Ongoing follow-up and imaging are important for managing chronic cases and preventing complications [8].

## Conclusions

Differentiating between sarcoidosis and TB involves a combination of clinical evaluation, radiographic assessment, histological analysis, microbiological testing, and advanced diagnostic assays. The similarities in symptoms and imaging findings make accurate diagnosis challenging. Still, a thorough and multi-faceted approach is essential for distinguishing between these two conditions and ensuring effective treatment, as they differ for both. Effective management involves tailored treatment plans and diligent monitoring to improve patient outcomes.
